# Solitary skull metastasis as initial manifestation of hepatocellular carcinoma

**DOI:** 10.1186/1477-7819-6-66

**Published:** 2008-06-21

**Authors:** Yu Shik Shim, Jung Yong Ahn, Jun Hyung Cho, Kyu Sung Lee

**Affiliations:** 1 Department of Neurosurgery, Yonsei Brain Research Institute, Yonsei University College of Medicine, Seoul, Republic of Korea; 2 Department of Neurosurgery, Sacred Heart Hospital, Hallym University College of Medicine, Chuncheon, Republic of Korea

## Abstract

**Background:**

A solitary skull metastasis from hepatocellular carcinoma (HCC) prior to diagnosis of the primary tumor without liver dysfunction is a very rare event.

**Case presentation:**

A 71-year-old male, without known liver disease, presented to our institution with a palpable occipital scalp mass. On brain magnetic resonance imaging (MRI), a highly enhanced and osteolytic skull tumor was observed. The histological diagnosis obtained from the percutaneous needle biopsy was a cranial metastasis from HCC. The metastatic tumor was removed via occipital craniectomy, and the two primary liver mass lesions were subsequently treated by transarterial chemoembolization.

**Conclusion:**

An isolated skull metastasis may be the sole initial presentation of HCC. Early diagnosis is essential in order to treat the primary disease. A skull metastasis from HCC should be considered in the differential diagnosis in patients with subcutaneous scalp mass and osteolytic defects on X-ray.

## Background

Hepatocellular carcinoma (HCC) is the fifth most common cancer in the world and is especially prevalent in African and East Asia [1]. The higher incidence of HCC in Asia is related to the increased prevalence of chronic viral hepatitis B [2]. Late-stage HCC usually metastasizes to the regional lymph nodes and lungs [3], but less commonly to the skeleton. HCC usually metastasizes preferentially to the vertebral column, pelvis, and ribs, but rarely to the skull [4].

Although the incidence of bone metastases in HCC has been described as very low in autopsy studies, an increasing trend has been reported recently [4,5]. In the past, because of its short survival of patients with HCC, their clinical presentations were mostly concerned with the manifestations of the primary cancer itself. However, recent progress in the treatment of HCC has made it possible for the patient to survive longer, and as a result, distant metastasis from HCC, including bone metastasis, has increased and attracted more attention than before [4].

In this report, we describe a patient without previously known liver disease who presented with metastatic HCC of the skull before the diagnosis of a primary cancer.

## Case presentation

**Figure 1:**
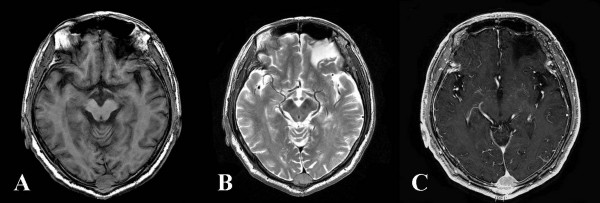
**Magnetic resonance imaging (MRI) of the skull metastasis from hepatocelluar carcinoma.** A) T1-weighted MRI and B) T2-weighted MRI demonstrating a homogeneous, well-defined, and iso-signal intensity mass in the occipital midline. C) Gadolinium enhanced T1-weighted MRI images showing a strong enhancement of the tumor.

**Figure 2:**
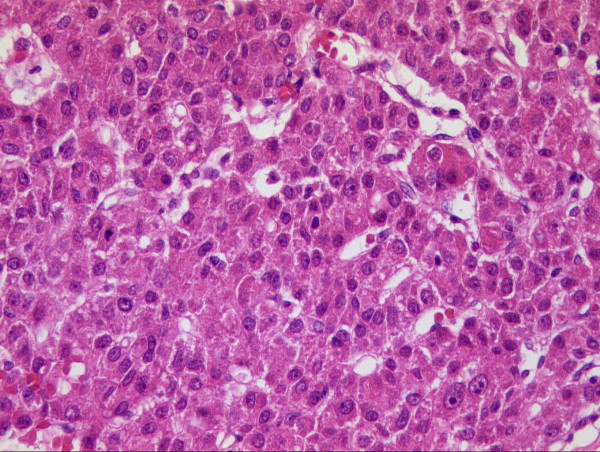
Hematoxylin-eosin staining (400× magnification) showing a pleomorphic tumor cell with eosinophilic cytoplasm and prominent nucleoli arranged in both a trabecular and solid pattern, which is consistent with metastasis of HCC.

**Figure 3:**
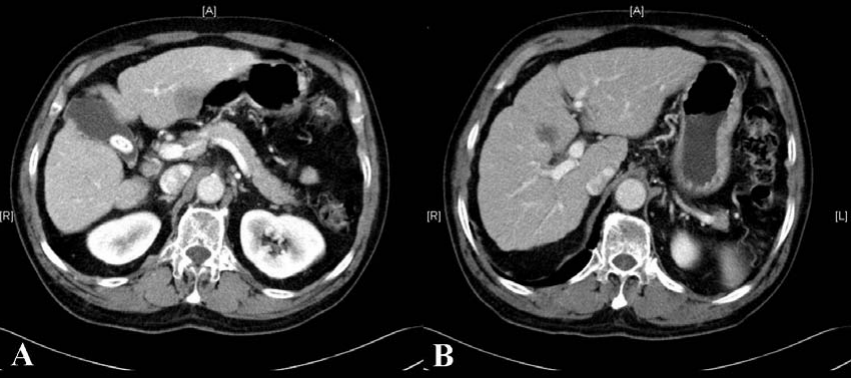
Abdominal computed tomography (CT) revealing two separate, low-density, 2-cm masses on segment II (A) and IV (B) of the liver.

A 71-year-old male presented with a scalp mass found incidentally one month prior to presentation. He did not have a history of a recent head trauma or of any significant medical problems, including liver disease. However, he had consumed seven alcoholic beverages (distilled liquor) per week for the last 20 years. On admission, neurological and physical examination revealed no neurological deficits or hepatomegaly. A soft and non-movable mass about 3 × 4 cm over his occipital area was noted. The mass was slowly growing and caused occasionally mild regional tenderness. Laboratory tests demonstrated normal liver function test, an alpha-fetoprotein level of 2.5 ng/ml and a positive serologic test for hepatitis C-virus (HCV) antibody. Identifiable risk factors for HCV infection such as intravenous drug use, multiple sexual partners, blood transfusion and receipt of blood-clotting factors were not revealed in this patient. Polymerase chain reaction to detect HCV RNA in the serum was not performed. X-ray film of the skull showed an osteolytic skull mass over the occipital midline area. On brain MRI, the tumor was a homogenous, well-defined mass with involvement of the inner and outer skull table. It revealed iso-signal intensity on T1-weighted (Figure 1A) and T2-weighted imaging (Figure 1B), with strong enhancement by Gadolinium (Figure 1C). A percutaneous needle biopsy showed pleomorphic tumor cells with eosinophilic cytoplasms and prominent nucleoli arranged in both a trabecular and solid pattern, findings that are consistent with metastasis of HCC (Figure 2). A retrograde diagnostic work-up for detecting the primary cancer was performed. An abdominal CT scan revealed two separate, low-density, 2-cm hepatic masses in segments II and IV of the liver (Figure 3). An abdominal sonogram identified nodular liver suggestive of cirrhosis. A total skeletal bone scan and contrast-enhanced chest CT scan revealed no other lesions.

**Figure 4:**
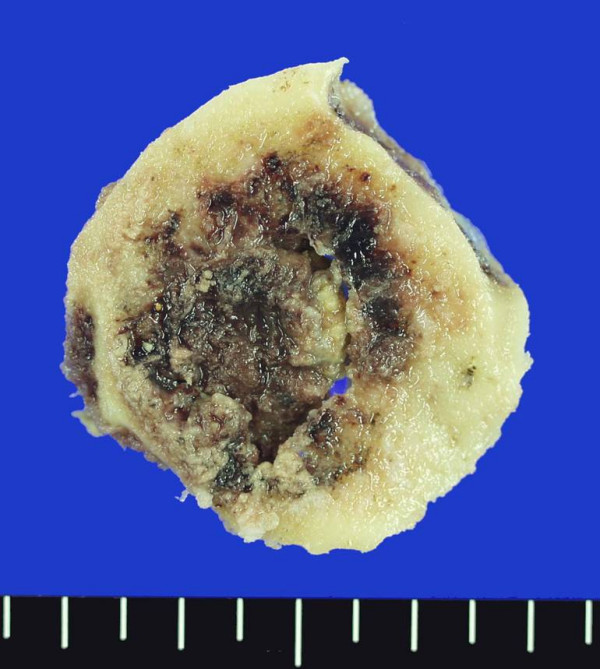
A grossly well-demarcated, reddish-brown tumor (reflecting high vascularity) penetrated both tables of the skull through the diploic space.

Under general anesthesia, the tumor was radically resected with surrounding normal bone via occipital craniectomy. At the time of surgery, a grossly well-demarcated reddish-brown mass (reflecting its high vascularity) penetrated both tables of the skull through the diploic space (Figure 4). The underlying dura matter was intact and did not show evidence of gross tumor-invasion. The dural surface attached to the tumor was curetted and cauterized by the bipolar forceps. To treat the primary cancer, transarterial chemoembolization with adriamycin (30 mg), lipiodol, and Gelfoam particles was performed after selecting a feeding artery of the tumor on aortography.

The patient was still alive after 9 months of follow-up without hepatic dysfunction due to the progression of primary cancer. He also did not show any evidence of the recurrence of skull metastasis.

## Discussion

The incidence of skeletal metastasis from HCC is estimated to be 2%–16%, depending on the prevalence of the primary disease in the population [4,6]. The most frequent sites of osseous metastases from HCC are vertebrae, the sternum, ribs, and long bones, although the incidence in the skull is low at 0.5%–1.6% [1,7-9]. Skull metastases from HCC predominantly affect males in their sixth and seventh decades, with similar age and sex distributions to those with only HCC [9].

In spite of the low incidence of skull metastases, there have been interesting trends in the reports about skull metastases from HCC. Moreover, in comparison with the incidence of skull metastases before the 1980's, the incidence after the 1990's has clearly increased because of a prolonged survival rate due to recent progress in the diagnosis and treatment of HCC [4,7]. Therefore, particularly in Asia, patients with HCC should be closely monitored for skull metastases. Plain skull x-ray is the most frequent initial diagnostic step in the patient with clinical suspicion of a bone lesion and bone scan with technetium-99^m^-methylene diphosphonate is widely used as a screening tool to detect bone metastases. On radiological examination, osteolytic-type behavior with a tendency to be highly enhanced is the most common finding [6,7]. However these findings are not specific to just HCC: most skull metastases appear as osteolytic, expansile, and hypervascular lesions [10]. Metastases are the most common cranial neoplasms in adults, of which 60% are from breast and lung carcinoma, although hematogenous skull metastases can be caused by nearly all types of tumors [11].

Because of the wide spectrum of primary cancers causing skull metastases, and because of their indistinguishable radiological findings, pathological confirmation by biopsy is required. Our case also required a biopsy for histopathological confirmation of the skull tumor. Metastases from HCC seldom emerge as a first diagnosis of a solitary skull tumor, because skull metastasis from HCC prior to diagnosis of the primary cancer is very rare [7]. Moreover, our patient had a normal liver function test and a normal alpha-fetoprotein and was asymptomatic from his primary liver cancer. A solitary skull metastasis from HCC is very rare, but this type of metastases could be explained by the osseous route of HCC metastasis.

Metastases in the central nervous system from HCC generally occur through two different kinds of pathways in the advanced stage of HCC [6,7]. One of them is the hematogenous route via the lung to the brain parenchyma without skull involvement. In this group, the character of HCC is defined as a "neutrophilic" cancer, and the lung is the most common site of extracranial metastases. On the other hand, the second route is the osseous route via Batson's venous plexus to the skull. In this group, bone is the most common site of extracranial metastases, and HCC is characterized as an "osteophilic" cancer. Cancer cells might disseminate within the dipole via the diploic venous channels and expand through the inner and outer table of the skull [7]. Therefore, it is difficult to find the skull metastasis from HCC without the presence of other bone metastases.

This patient initially visited the hospital due to the incidentally discovered scalp mass. In the literature, a subcutaneous mass with occasional pain is the most common clinical presentation (63%), followed by neurological deficits (44%), headache (11%), and seizure [1]. The neurological deficits, such as facial palsy, deafness, visual disturbance, facial numbness, weakness of limbs, and other cranial nerve palsies, are associated with the tumor size and location. Neurological deficits are more frequently present in metastases of the skull base rather than the cranial vault [1,10].

Several treatment options can be used to treat the skull metastases from HCC, including direct ethanol injection therapy, radiotherapy, surgical resection, and supportive management. Many previous reports suggest that most patients with skull metastases died due to liver failure and that surgical resection of the metastatic lesion could not prolong survival [1,4,10]. The stage of the primary cancer is mostly associated with the prognosis. However, surgical resection of the skull metastases is acceptable for preventing intracranial hemorrhage and neurological deterioration. Surgical intervention also allows for biopsy, whereby the tumor can be histologically confirmed; such biopsies are easier to perform in cranial vault-sited cases [1,3,12].

## Conclusion

Although a solitary skull metastasis prior to the diagnosis of HCC demonstrates rare metastatic behavior for HCC, especially in Asia, skull metastases from HCC should be included in the differential diagnosis of skull tumors, even if the patient is asymptomatic of liver cirrhosis. With the increase of survival in HCC patients, clinically significant bone metastases have also increased, affecting the patients' quality of life. Therefore, early diagnosis and proper management of bone metastasis from HCC is essential to prevent deterioration in the quality of life of HCC patients.

## Competing interests

The authors declare that they have no competing interests.

## Authors' contributions

YSS conceptualized the study, gathered the data, and drafted the manuscript, JHC performed the literature search and helped to draft the manuscript, JYA supervised the process and finally approved the manuscript for publication, KSL was involved in manuscript revision. All authors have read and approved the final manuscript.
